# Cyberbullying and mental health: past, present and future

**DOI:** 10.3389/fpsyg.2023.1279234

**Published:** 2024-01-15

**Authors:** Suhans Bansal, Naval Garg, Jagvinder Singh, Freda Van Der Walt

**Affiliations:** ^1^University School of Management and Entrepreneurship (USME), Delhi Technological University, New Delhi, India; ^2^Department of Operational Research, University of Delhi, New Delhi, India; ^3^Faculty of Management Sciences, Department of Business Management, Central University of Technology, Bloemfontein, South Africa

**Keywords:** cyberbullying, mental health, bibliometric analysis, cyber perpetration, cyber victimization

## Abstract

**Purpose:**

Cyberbullying has attracted the world's attention, and therefore researchers across the world have contributed to the literature on cyberbullying and mental health. Amongst others, they have conducted bibliometric analyses and associated cyberbullying with various factors but have not determined the impact of cyberbullying on people's mental health. Hence, the aim of this study was to conduct bibliometric analyses of cyberbullying and mental health to analyze the academic performance of the literature on impact of cyberbullying on people's mental health; and to propose future research avenues to make further contributions to this field of study.

**Methodology:**

Spreadsheets and VOSviewer were used to conduct the bibliometric analysis. The data were extracted from the SCOPUS database which provided an extensive collection of data and journals.

**Findings:**

Having explored the top active countries publishing on the impact of cyberbullying on people's mental health and the academic performance of such research articles by means of a qualitative bibliometric analysis, the results revealed that this research topic is still to be researched extensively. The study also suggests countries/regions where this research topic can be explored further, as well as possible journals for publication of research results, and further studies to be conducted.

**Discussion:**

The literature presents a fragmented view on the impact of cyberbullying on people's mental health. Studies on cyberbullying are limited for the reasons as discussed in this article. Hence, bibliometric analysis was conducted to analyze the performance of academic literature on the impact of cyberbullying on people's mental health; the academic performance of research articles on cyberbullying and mental health; and to make proposals toward a future research agenda.

## Introduction

Mental health has garnered significant attention from the research community, academics, and policymakers across the globe (Somé et al., 2022), and has emerged as a major contributor to the global health crisis (Wang et al., 2021). The World Health Organization (WHO) defines mental health as “a state of wellbeing in which the individual realizes his or her abilities, can cope with the normal stresses of life, can work productively and fruitfully, and is able to make a contribution to his or her community” (World Health Organization, 2004). According to the definition, a mentally healthy person effectively manages stress, work to his/her optimal output levels, and positively contribute to his/her community. The definition also suggests that the absence of, or an impaired state of mental wellbeing may hinder individuals from realizing their full potential, hamper their productivity, and diminish their ability to contribute positively to their communities (Somé et al., 2022). The WHO report on mental health reveals that nearly one in every eight individuals globally experience mental health issues (World Health Organization, 2019). The report further indicates that depression is one of the major factors contributing to impaired mental health, affecting almost 3.8% of the world's population (World Health Organization, 2022), including 5% of adults, with the rest being children and adolescents (World Health Organization, 2022). Several factors such as genetics (Shabani et al., 2019), environment (Usher et al., 2019), unhealthy lifestyle choices (Lim et al., 2016), extreme conditions such as COVID-19 (Greenberg, 2020; Moreno et al., 2020), broken relationships and lack of social support (Mehtaa et al., 2023), excessive usage of social media (Karim et al., 2020), and bullying experiences (Giumetti and Kowalski, 2022) contribute to the rising mental health issues across the globe. People of all ages, professions, genders, geographic regions, colors, castes, and creeds suffer from mental health issues (Oksanen et al., 2020; Achuthan et al., 2023; Bansal et al., 2023a,b). Furthermore, there has been a significant rise in mental health issues since late 2019 due to the COVID-19 pandemic and the resultant exponential increase in internet usage. Although COVID-19 is on the decline, the negative repercussions of high internet use are still visible. One of its most annoying and unfortunate consequences is cyberbullying (Xie et al., 2020).

Cyberbullying is defined “as an aggressive, intentional act carried out by a group or individual, using electronic forms of contact, repeatedly and over time against a victim who cannot easily defend him or herself” (Smith et al., 2008, p. 376). In other words, cyberbullying refers to an intentional and repetitive act carried out using electronic media or information communication technology (ICT) to bully an individual or group who is defenseless against these attacks. This type of bullying differs from traditional bullying in various ways. ICT allows bullies to hide their identities and bully others as often as they want to (Bashir Shaikh et al., 2020). Hashemi (2021) also differentiate cyberbullying from traditional bullying, suggesting that cyberbullies can bully a large number of victims at any given point in time. They further suggest that cyberbullying may leave long-lasting memories in its victims' minds, also known as a digital footprint (Hashemi, 2021). It takes diverse forms in different situations. For instance, flaming occurs when a perpetrator uses foul and violent language during online communication (Maichum et al., 2016), and trolling involves taunting a person or a group in a humorous but undignified manner (Zsa Tajol Asanan, 2017). Denigration involves spreading malicious information to damage a victim's reputation (Zainudin et al., 2016). Masquerading is pretending to be someone else, usually the victim (Peled, 2019). Some other modern forms of cyberbullying include outing and cyberstalking (Wright, 2018; Peled, 2019).

Although cyberbullying is a dreadful act, its adverse impact on an individual's physical and mental health necessitates an in-depth understanding of this phenomenon. Rao and Rao (2021) are of the view that cyberbullying may result in the development of mental health issues, depression (Englander, 2021), anxiety, psychological distress, and post-traumatic stress symptoms (Nochaiwong et al., 2021). The events of cyberbullying are traumatizing and psychologically wounding (Paat and Markham, 2020). Victims of cyberbullying may develop depressive symptoms and insomnia (Kim et al., 2020), and counterproductive work behavior, along with experiencing lower job satisfaction levels (Kowalski et al., 2017). Victims may also show lower engagement (Muhonen et al., 2017) and higher attrition intentions (Li et al., 2018). Students are among the worst affected victims (Kowalski and Limber, 2013). They suffer from negative consequences such as higher absenteeism, lack of concentration (Kowalski et al., 2018), feelings of shame and guilt (Ciucci and Baroncelli, 2014), and engaging in anti-social behavior (Cavalcanti et al., 2019). Maurya et al.'s (2022) 3 year longitudinal study reported that the rates of cyberbullying had increased from 3.8% to 6.4% in female and from 1.9% to 5.6% in male respondents over study's period. Also, their study suggested that female respondents have developed a high rate of suicidal ideation compared to male participants due to experiencing cyberbullying. Furthermore, Xia et al. (2023) report that cyberbullying was one of the major reasons for the development of appearance anxiety in the college students, which has further exaggerated the social anxiety in them. The authors further reported that the combined effect of cyberbullying and appearance anxiety has caused higher social anxiety levels in the college students. Additionally, a study from Bangladesh revealed that university students who experienced cyberbullying during their tenure at the university had developed anger issues, self-guilt, and fear of attending college (Sheikh et al., 2023). Likewise, a study on Malaysian youth revealed that victims of cyberbullying had developed anxiety, stress, and exhaustion, which have resulted in an increase in suicidal ideations among them. Therefore, the rising literature on cyberbullying and mental health has necessitated to analyze its academic performance. Also, acknowledging the importance of review and bibliometric studies, several contemporary researchers have suggested that bibliometric studies on cyberbullying and mental health issues should be conducted.

A bibliometric analysis is a research method involving the analysis of published literature to identify patterns and trends in a particular field (Donthu et al., 2021). Its applicability is multidisciplinary (Andersen, 2019), with various researchers having conducted bibliometric analyses in fields such as human resources (Andersen, 2019), journalism (Bansal et al., 2023a), corporate governance (Singhania et al., 2022a,b) and ecopreneurship (Guleria and Kaur, 2021). This study used bibliometric analysis to analyze the research documents on cyberbullying and mental health. Further, Donthu et al. (2021) have suggested using bibliometric analysis over meta-analysis and structured literature review based on the following differences. Firstly, the scope of the study is broader, and the goal is to summarize vast amounts of bibliometric data for presenting the performance and state of the academic intellect. Secondly, the aim is to analyze the emerging trends of the field. Thirdly, the amount of literature is too large for manual reviewing and the analysis requires a mix of qualitative and quantitative analyses [for detailed comparison, please refer to the Table 1 of Donthu et al. (2021)]. Therefore, bibliometric analysis could provide insight into the extent and scope of research on cyberbullying and its impact on mental health by identifying top influential articles, journals, and authors, as well as identifying gaps in the literature and potential areas for future research through topical analysis and analyzing new emerging keywords and topics. Consequently, bibliometric analysis was considered the best choice for analyzing the past, present and future of cyberbullying and its impact on the mental health.

Moreover, bibliometric analysis on cyberbullying and mental health have not been conducted extensively. Saif and Purbasha (2023) performed a qualitative systematic literature review on young females in developing countries who have experienced cyberbullying. They differentiated and categorized the instances of cyberbullying those young females faced. Additionally, the bibliometric analysis of Achuthan et al. (2023) focused on studies on cyberbullying and sustainable development and the impact of COVID-19 on this relationship. Shao and Cao (2021) analyzed the existing literature on adolescent cyberbullying retrieved from the Web of Science. Furthermore, Barragán Martín et al. (2021) focused on analyzing literature on cyberbullying from adolescents' perspective published in the Web of Science database, without specifically focusing on mental health. Moreover, the bibliometric analysis conducted by Cretu and Morandau (2022) focused on education setups and cyberbullying in relation to adolescents. Peker and Yalcin (2022) focused on studying cyberbullying literature published in the Web of Science database only. However, their study is limited to studies published up until 24 July 2021, and only addresses topics such as cooperation between countries, institutions, and authors. They did not analyze keywords or identify emerging trends in cyberbullying literature. Their study also did not study the relation between cyberbullying and mental health. Other studies have focused on victims of cyberbullying (e.g., Mäntylä et al., 2018; López-Meneses et al., 2020; Gómez Tabares and Correa Duque, 2022), educational setting (Moreno and Piqueras, 2020), or have been location-specific, such as those conducted in Turkey (Manap, 2022), Spain (Andrés et al., 2016) and Latin America (Herrera-López et al., 2018; Villanueva et al., 2020). Finally, Kim et al. (2021) studied literature focusing on workplace cyberbullying in medical and hospital setups. Therefore, our study will cover the highlighted gap and present a fresh perspective on the impact of cyberbullying on the mental health.

### Motivation of the present study

This study differs from previous studies on two broad bases. Firstly, the preceding section presented fragmented studies found in the literature. Although previous studies have explored various aspects of cyberbullying, they have their own limitations, such as being limited to location, age group, or even sustainable development topics. Some studies fail to address the relationship between cyberbullying and mental health, or only focus on a particular age or a specific group involved in cyberbullying. Secondly, previous studies have primarily utilized systematic literature reviews to explore the nexus of cyberbullying and mental health. This study aims to address these limitations by conducting a comprehensive analysis of the global literature on cyberbullying and mental health. The study was not confined to a specific geographic location, age group, or work setting. Instead, the aim was to explore the past, present, and future of the knowledge that pertains to the impact of cyberbullying on people's mental health. Thirdly, mental health is a serious concern which has attracted global attention. According to various researchers many factors can be associated with degradation of mental health, including cyberbullying (Schodt et al., 2021). Also, as discussed in the preceding section, cyberbullying can negatively affect mental health like developing depressive symptoms in the victims (Kowalski et al., 2022), suicidal ideation (Kowalski and Limber, 2013), stress and anxiety (Nochaiwong et al., 2021; Rao and Rao, 2021). Hence, it has become imperative to study effects on cyberbullying on the mental health. Also, with the help of present study we can analyze the academic performance of the literature on the topic under consideration and discuss certain future trends. Additionally, the SCOPUS database was utilized, which has not been extensively used in previous bibliometric studies on this topic. This database was chosen due to its various merits, as will be discussed in the forthcoming sections. Furthermore, a bibliometric approach was adopted, which helps mitigate the risk of bias that can be generated during a systematic literature review. Unlike a systematic literature review, a bibliometric analysis includes all the identified studies on the topic that fall within its scope. Lastly, the current study seeks to address the following:

(a) What are present trends in research publications, citations, and research areas?
(b) What is countries' performance and authors' performance on the topic?
(c) How are the journals performing on the said topic?
(d) How are articles performing? and
(e) What are widely used keywords and emerging topics?

Apart from a general study of these topics, this study explored (a) emerging countries and countries where research on the topic has not been done yet, (b) emerging journals, and (c) top publications on the impact of cyberbullying on people's mental health published during the past 5 years. Consequently, the authors systematically extracted and explored literary data (research publications) from the SCOPUS database. This study finds its design on the basis of suggestions and guidelines provided by Donthu et al. (2021). Lastly, software such as spreadsheets and VOSviewer were used to analyze the final dataset.

## Methodology

### Database selection

The SCOPUS database was used to extract data for bibliometric analysis. SCOPUS is the largest database of peer-reviewed scientific literature including journals, books, and conference proceedings. Other databases such as Google Scholar, Pubmed, PsycInfo, and Web of Science (WoS) are also available, but the SCOPUS database was used as it rendered results more relevant to the context of this study. Google Scholar does not provide a dataset in a format suitable for bibliometric analysis. The PubMed dataset predominantly comprises research in life sciences and biomedical topics, whilst the PsycInfo database consists of works in the field of psychology. However, cyberbullying studies are covered under several themes such as psychology, human resources, economics, medical, education, sociology, etc. Also, SCOPUS is the largest database and it provides information regarding the most prominent authors, countries, affiliations, journals, and publication years both in tabular and graphic form. Therefore, the SCOPUS database is preferred over all other available databases.

### Data extraction

Previous researchers used terms such a “cyberbullying,” “cyber bullying,” “mental health,” “bully,” “victimization,” “perpetration,” and “bystander” in their studies. In the present study, terminology such as perpetrators or victims were not used, as it would have restricted the study to exploring mental health issues experienced either by victims or perpetrators, only. Moreover, based on our previous bibliometric study on cyberbullying, it was found that (a) no bibliometric study had analyzed the literature on the nexus of cyberbullying and mental health and (b) mental health is one of the major fields of study in collaboration with cyberbullying (Bansal et al., 2023a). Although various authors have used a vast list of keywords while retrieving a dataset on mental health (Guo et al., 2023), they have skipped many terms; for instance, “suicidal,” “suicidal tendency,” “suicide,” “suicidal ideation,” and “deviant behavior.” Also, it was observed during our extensive literature review, apart from specifying mental disorder, the term “mental health” was also used as one of the keywords or extended keywords. Therefore, we have used “mental health” as an umbrella term to not to skip any study that might have explored any one mental health problem arising out of cyberbullying. Since the objective of the study was to analyze academic knowledge on cyberbullying and mental health from both the victims' and perpetrators' perspectives, “cyberbullying” OR “cyber bullying” AND “mental health” were used in the search string. Consequently, 628 documents were retrieved.

### Inclusion and exclusion criteria

Results that contained 'cyberbullying,' 'cyber bullying' and 'mental health' in the title, abstract and keywords were deemed fit for the study. Documents published only in English until 31 December 2022 were considered as publications for year 2023 were over during the data exploration time frame and time frame limiting is based on the recommendations of various researchers including Singh et al. (2021) and Donthu et al. (2021). Boolean 'OR' was used to maximize the results, whereas 'AND' was used to limit the research results to mental health issues. Based on this inclusion and exclusion criteria, 576 articles met the requirements. Documents such as book titles and conference papers were excluded, as they are not always subjected to a rigorous peer-review process (Singh et al., 2021). This resulted in the exclusion of 108 documents. Finally, 468 documents were considered for the data screening process.

### Data screening

**Figure 1:**
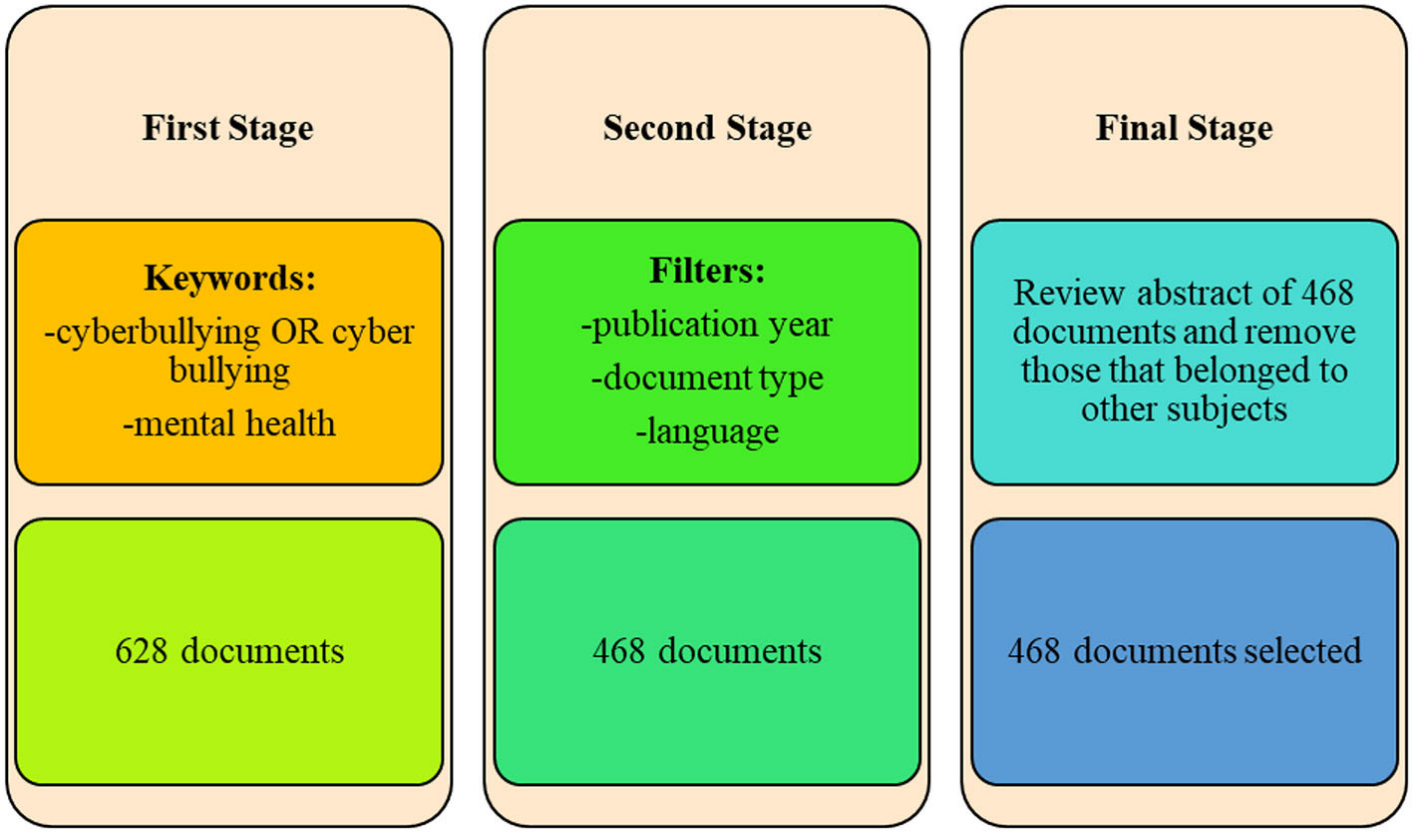
Search model. Source: SCOPUS, Authors' creation.

The authors studied the abstracts of 468 documents and found that they all met the criteria. Figure 1 presents the search model.

### Data analysis

Spreadsheets and VOSviewer were used to analyze the data obtained from the SCOPUS database. Specifically, descriptive statistics were applied to generate various tables and charts to explore patterns in the data. These charts and tables aided in the analysis of publications and citation trends. VOSviewer was employed to analyze the most influential articles, journals, and authors, rank nations based on their publications and citation activity, and to perform keyword analysis and evolution. The study also examined the evolution of academic knowledge on the subject across different countries, authors, and journals. Citation and co-citation analyses in VOSviewer were utilized to conduct the aforementioned analyses, as suggested by previous researchers such as Singh et al. (2021) and Donthu et al. (2021).

## Results and discussion

### Publication and citation trends

**Figure 2:**
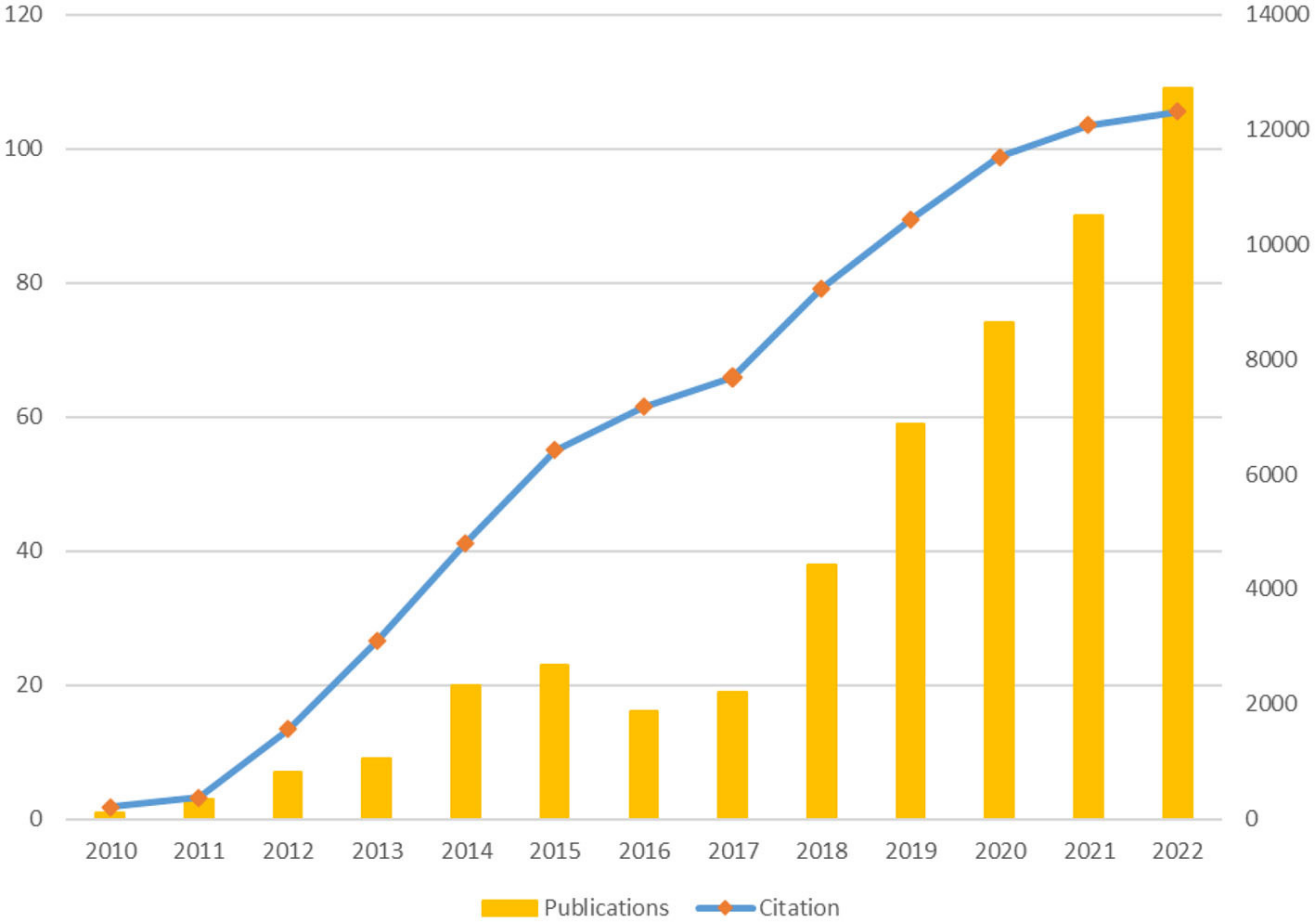
Publication and citation trend. Source: SCOPUS database.

For purposes of this study, 406 articles and 62 reviewed articles were studied. Annual publications on the impact of cyberbullying on people's mental health remained under 10 until 2013, reaching 30 by 2017. In 2018, the annual publications surpassed 50, and in 2022, they exceeded 100. In the last 5 years (2018–2022), there were 370 publications on this topic, accounting for 79.05% of total publications during the thirteen-year period. Over a period of time, the number of articles and review articles increased from 113 to 324, and from 23 to 46, respectively. Furthermore, there were 12,322 citations from 2010 to 2022, with an average of 26.33 citations per publication. Citations from the last 5 years constituted 37.62% of the total citations. Figure 2 illustrates the publication and citation trends in the field of cyberbullying and mental health research, depicting a steady rise in both publications and citations. The earliest document, Wang et al.'s (2010) “Co-occurrence of Victimization from Five Subtypes of Bullying: Physical, Verbal, Social Exclusion, Spreading Rumors, and Cyber,” examined the co-occurrence of subtypes of peer victimization. It assessed victimization related to verbal, spreading rumors, social exclusion, and physical and cyberbullying. The study also found that male participants were more likely to become victims of all the subtypes of bullying.

### Journal analysis

Table 1 presents the top 10 journals based on the number of documents published. Out of 187 journals that have published at least one article on cyberbullying and mental health, only four journals had 10 or more publications on these topics. The *International Journal of Environmental Research and Public Health* had the highest number of publications (67) and citations (703). It is a multidisciplinary journal covering public health, occupational health, and psychology, and is published by MDPI. It is followed by the *Journal of Adolescents Health* with 12 publications and 2,016 citations. This journal is published by Elsevier and focuses on domains such as medicine, pediatrics, and public health. The third-ranked journal is the *Children and Youth Services Review*, with 535 citations. It is also published by Elsevier and has a multidisciplinary approach, primarily focusing on sociology, political sciences, and education. The majority of the top 10 journals publish multidisciplinary works, with an emphasis on behavioral sciences, social sciences, and adolescents, indicating the significance of cyberbullying and mental health in these fields. The only journal with more than 1 000 citations is the *Journal of Adolescents Health* (2 016 citations). It also has the highest citations per document score of 168. The journal with the second-highest citations per document score is the *Children and Youth Services Review* (89.17 citations per document). Lastly, it is worth noting that only a few studies on cyberbullying and mental health have been published in journals from renowned publishers such as the American Psychological Association (two publications), Taylor and Francis Ltd. (two publications), and Oxford University Press (one publication).

**Table 1:** Top 10 most active journals.

**Journal**	**Documents**	**Citations**	**Publisher**	**SCOPUS cite score**
International Journal Of Environmental Research And Public Health	67	703	*MDPI*	4.5
Journal of Adolescent Health	12	2,016	*Elsevier*	7.1
Cyberpsychology, Behavior, And Social Networking	12	203	*Mary Ann Liebert*	6.7
Journal Of Interpersonal Violence	10	154	*Sage*	4.5
Computers In Human Behavior	9	302	*Elsevier*	1.2
Plos One	8	338	*Public Library of Science*	5.6
Children And Youth Services Review	6	535	*Elsevier*	3.3
International Journal Of Public Health	5	254	*Frontiers Media*	5.3
Journal Of School Health	5	215	*Wiley Blackwell*	3.3
Journal Of Child And Adolescent Trauma	5	141	*Springer Nature*	2.9

Source: SCOPUS, VOSviewer.

**Figure 3:**
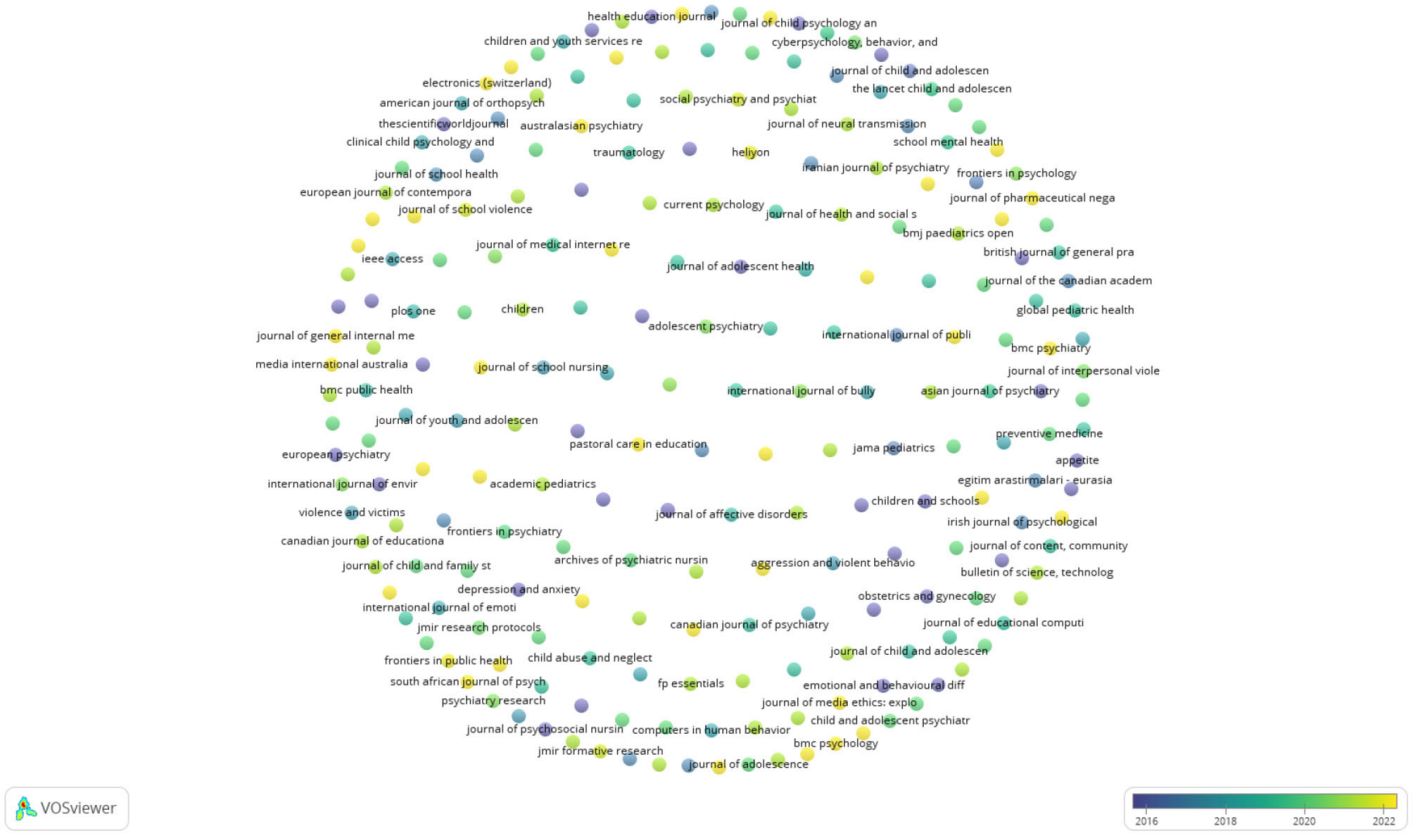
Journal overlay map. Source: SCOPUS, VOSviewer.

A journal overlay visualization (Figure 3) was conducted to explore the emerging journals and the earliest journals published in the field. Another aim of journal overlay analysis was to determine the shift in research areas that journals publish on. The journals marked in yellow color is emerging journals in this field, whereas those marked in bluish-purple are the earliest journals published in the field. Journals such as the *American Journal of Health Promotion, Australian Psychiatry, BMC Psychology*, and *Frontiers in Digital Health* are emerging journals in the field, to name a few.

### Top cited publications

The top 10 highly cited research articles in the SCOPUS database are presented in Table 2. One of these articles, “Psychological, physical, and academic correlates of cyberbullying and traditional bullying,” authored by Kowalski and Limber (2013), aimed to analyze the relationship between experiences of cyberbullying and traditional bullying in children and adolescents, and their psychological and physical health as well as academic performance. They conducted a survey comprising of variables such as experiences with traditional bullying and cyberbullying, depression, anxiety, physical wellbeing, and academic performance (Kowalski and Limber, 2013). Participants were divided into four cohorts: cyber victims, bullies, victims/bullies, and not involved in cyberbullying. A similar division was also made for traditional bullying. It was found that students in the victims/bullies' group had the worst scores for their psychological and physical health and academic performance, especially among male participants. In comparison, female participants were more likely to develop both traditional and cyberbullying-related anxiety. The results further highlighted that although cyber victimization and perpetration and traditional bullying victimization and perpetration positively correlated with anxiety, depression, lower self-esteem, suicidal ideation, absenteeism and leaving school early, victimization in both types of bullying was more significantly associated with these outcomes than perpetration. In other words, the study emphasized that victims of traditional and cyberbullying are more likely to develop anxiety, depression, and suicidal ideation than perpetrators, especially in school learners.

**Table 2:** Top 10 most influential publications.

**Title**	**References**	**Journal**	**Citations**
Psychological, physical, and academic correlates of cyberbullying and traditional bullying	Kowalski and Limber, 2013	*Journal of Adolescent Health*	682
Cyberbullying, school bullying, and psychological distress: a regional census of high school students	Schneider et al., 2012	*American Journal of Public Health*	557
Online communication, social media and adolescent wellbeing: a systematic narrative review	Best et al., 2014	*Children and Youth Services Review*	492
Longitudinal and reciprocal relations of cyberbullying with depression, substance use, and problematic internet use among adolescents	Gámez-Guadix et al., 2013	*Journal of Adolescent Health*	337
Victims' perceptions of traditional and cyberbullying, and the psychosocial correlates of their victimization	Campbell et al., 2012	*Emotional and Behavioral Difficulties*	246
Cyberbullying: review of an old problem gone viral	Aboujaoude et al., 2015	*Journal of Adolescent Health*	245
Annual research review: harms experienced by child users of online and mobile technologies: the nature, prevalence and management of sexual and aggressive risks in the digital age	Livingstone and Smith, 2014	*Journal of Child Psychology and Psychiatry*	234
Prevalence and effect of cyberbullying on children and young people	Hamm et al., 2015	*JAMA Pediatrics*	216
Co-occurrence of victimization from five subtypes of bullying: physical, verbal, social exclusion, spreading rumors, and cyber.	Wang et al., 2010	*Journal of Pediatric Psychology*	211
Cyber bullying and physical bullying in adolescent suicide: the role of violent behavior and substance use	Litwiller and Brausch, 2013	*Journal of Youth and Adolescence*	195

Source: SCOPUS, VOSviewer.

The second most influential study is “Cyberbullying, School Bullying, and Psychological Distress: A Regional Census of High School Students,” authored by Schneider et al. (2012). The authors aimed to analyze the relationship between cyberbullying and school bullying victimization and psychological distress (Schneider et al., 2012). Results revealed that 59.7% of students who experienced cyberbullying were also the prey of school bullying, while 36.3% of students who experienced more school bullying were also victims of cyberbullying. Furthermore, results indicated that victims of both school and cyberbullying had significant associations with psychological distress. The tenth most influential publication, titled “Cyber bullying and physical bullying in adolescent suicide: the role of violent behavior and substance use” is authored by Litwiller and Brausch (2013). The authors analyzed the relationship between physical and cyberbullying victimization and suicidal tendencies in adolescents, considering mediating variables such as violent and sexual behavior. The study's findings suggested that both types of bullying are highly associated with suicidal tendencies and unsafe sexual and violent behaviors. Additionally, the study highlighted an association between both types of bullying and substance abuse. Substance abuse and violent behavior acted as partial mediating variables. They explained “how risk behaviors can increase an adolescent's likelihood of suicidal behavior through habituation to physical pain and psychological anxiety” (Litwiller and Brausch, 2013, p. 675).

Table 3 illustrates the top five publications in the last 5 years, i.e., 2018 to 2022. At the top of the list is Arseneault's (2018) article titled “Annual Research Review: The persistent and pervasive impact of being bullied in childhood and adolescence: implications for policy and practice”. The article was published in the Journal of Child Psychology and Psychiatry and was cited 192 times. The study highlighted the impact of childhood bullying on mental and physical health as well as socio-economic outcomes. It demonstrated that childhood bullying, including traditional and cyberbullying, contributes to childhood and adolescent adjustment problems and may lead to poor mental and physical health, and the development of socio-economic difficulties. The study emphasized the importance of interventions against childhood bullying that focus on reducing symptoms-based problems in young victims. Furthermore, the study highlighted a need for developing individual level-based interventions to promote resilience against bullying behavior and reduce the risk of being vulnerable to bullying.

**Table 3:** Top five publications in the last 5 years.

**Title**	**References**	**Journal**	**Citations**
Annual research review: the persistent and pervasive impact of being bullied in childhood and adolescence: implications for policy and practice	Arseneault, 2018	*Journal of Child Psychology and Psychiatry*	192
Self-harm, suicidal behaviors, and cyberbullying in children and young people: systematic review	John et al., 2018	*Journal of Medical Internet Research*	185
COVID-19 racism and mental health in chinese american families	Cheah et al., 2020	*Pediatrics*	172
Mobile phone use and mental health. a review of the research that takes a psychological perspective on exposure	Thomée, 2018	*International Journal of Environmental Research and Public Health*	129
Roles of cyberbullying, sleep, and physical activity in mediating the effects of social media use on mental health and wellbeing among young people in England: a secondary analysis of longitudinal data	Viner et al., 2019	*The Lancet Child and Adolescent Health*	116

Source: SCOPUS, VOSviewer.

### Country/region analysis

**Figure 4:**
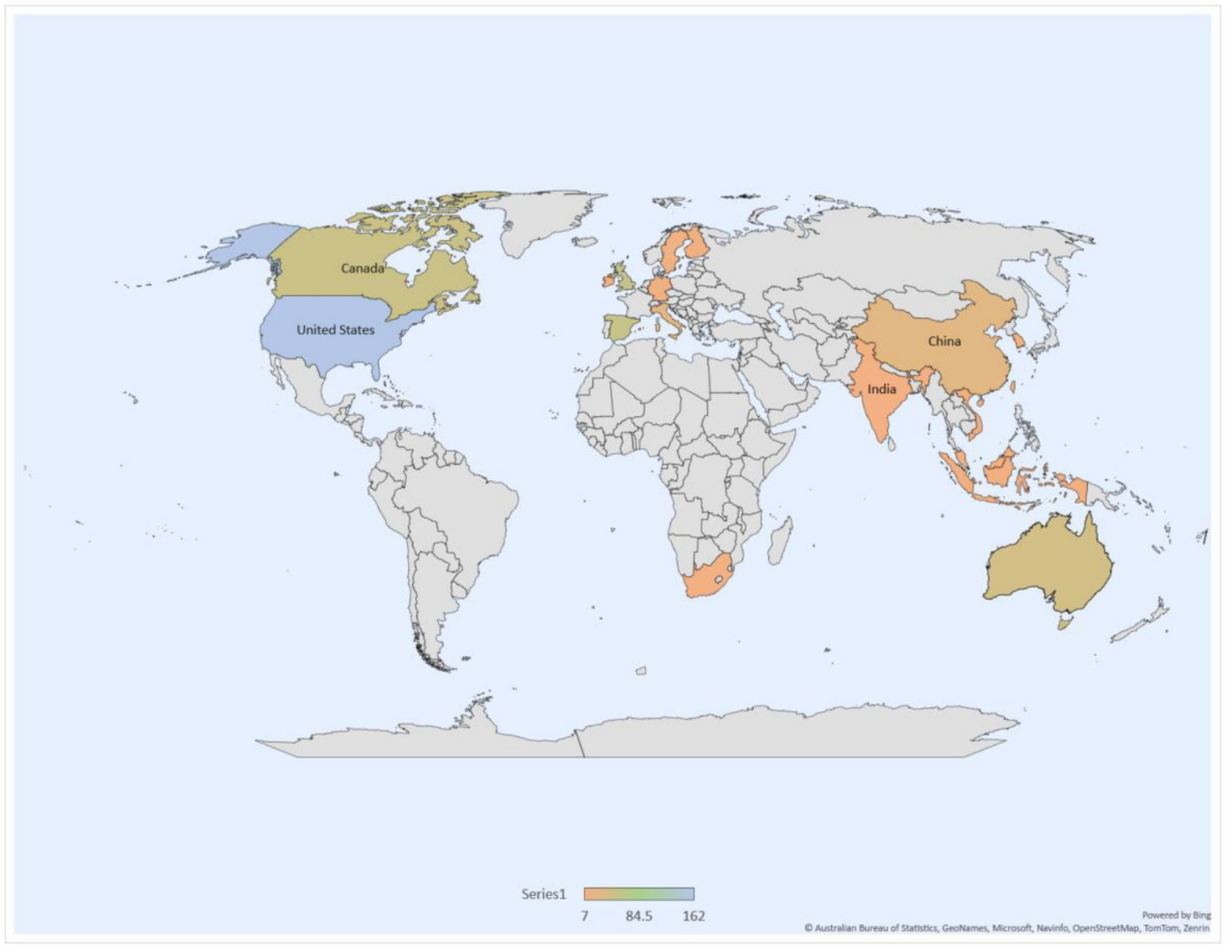
Country map. Source: SCOPUS database, VOSviewer.

**Figure 5:**
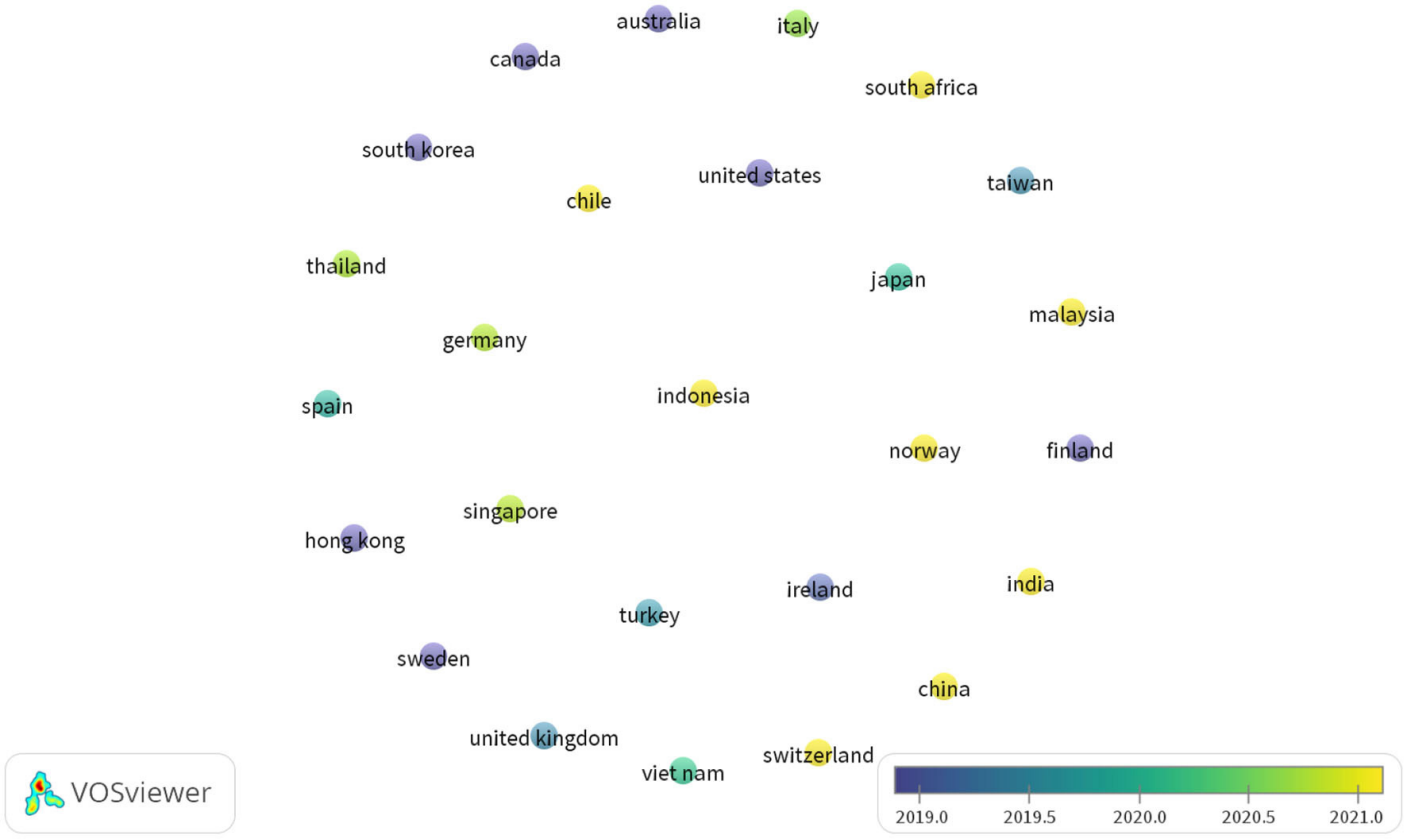
Country overlay map. Source: SCOPUS, VOSviewer.

Table 4 and Figures 4, 5 reports the top 20 countries/regions actively publishing in the cyberbullying and mental health domain. The SCOPUS database revealed that 75 countries have published 468 documents on this topic. The large number of countries involved suggests that cyberbullying and mental health have received global coverage and attention. It is interesting to note that 13 out of the top 20 countries are developed nations (Figure 4). The United States of America (USA) tops the list with 162 publications and 5 259 citations. It is followed by the United Kingdom (UK) with 55 publications and 2,045 citations. These two countries are developed economies that have been utilizing advanced technologies, especially ICT. Moreover, cyberbullying was recognized and addressed in developed countries earlier to any other country (Bansal et al., 2023a). Also, the most influential authors on cyberbullying are from developed nations. P. K. Smith tops the list of most influential authors with 454 citations, followed by S. Hinduja (346) and J. W. Patchin (320). China leads the list of most active developing nations with 25 publications and 380 citations. Indonesia follows China with eight publications and 60 citations, followed by Ireland (eight publications and 127 citations), Vietnam (eight publications and 91 citations), India (seven publications and 22 citations) and South Africa (seven publications and 31 citations). While the difference in the number of publications and citations between developing and developed economies is justified by the level of ICT technology used, it does not present a clear picture of the situation. Moreover, it is not justified to conclude that developing countries like India and China are laggers in research on the topic. Therefore, VOSviewer was used for purposes of this research to conduct a country overlay mapping to explore which are the emerging countries based on their paper publication year.

**Table 4:** Top 20 countries publishing on cyberbullying and mental health.

**Country**	**Documents**	**Citations**	**Economy**
United States	162	5,259	Developed
United Kingdom	55	2,405	Developed
Spain	54	1,111	Developed
Canada	49	1,340	Developed
Australia	41	1,342	Developed
China	25	380	Developing
Italy	22	244	Developed
Taiwan	21	333	Developed
Sweden	11	496	Developed
Finland	10	143	Developed
Germany	10	120	Developed
Indonesia	8	60	Developing
Ireland	8	127	Developing
South Korea	8	139	Developed
Vietnam	8	91	Developing
Hong Kong	7	120	Developed
India	7	22	Developing
Malaysia	7	12	Developing
Singapore	7	85	Developed
South Africa	7	31	Developing

Source: SCOPUS, VOSviewer.

The results of the country overlay mapping process categorize countries in five different colors. Dark bluish-purple presents the countries that have been active in this field for a very long time, whereas countries in yellow are the emerging countries. As expected, India and China are marked in yellow, suggesting a rise in research on cyberbullying and mental health in emerging countries. Interestingly, it also provides further insights, for example countries marked in dark bluish-purple color are countries with developed economies and with substantial literature based on the subject, as they have been publishing works for several years. On the other hand, countries marked in yellow represent developing economies that are emerging in this field. The ICT advances and the creation of early awareness regarding the impact of cyberbullying on mental health are a few differentiating factors between developing and developed economies. Figure 5 presents a country overlay analysis.

### Research area analysis

Table 5 presents the research areas in which studies on cyberbullying and its impact on mental health are being conducted. Medicine has the primary coverage with 36.9% of studies, followed by psychology with 19.1%, and social sciences (14.9%). Other major research areas include environmental science (9.0%), computer science (4.9%), arts and humanities (3.2%) and neuroscience (2.5%), to name a few. The results suggest that although most of the studies were conducted in the medical domain, there are cyberbullying and mental health researchers in other areas such as psychology, social sciences, environmental science, and computer science. Table 5 also reports a comparison between documents published in different research field in two-time frames, i.e. 2010–2018 and 2019–2022. It reports a percentage increase in the publication from 2010–2018 to 2019–2022 in research areas such as medicine, psychology, social sciences, environmental science, computer science, neuroscience, nursing, and engineering. It also reports a percentage decline in arts and humanities research areas during the same period. The table further illustrates the emergence of new research areas, namely business, management and accounting, energy, pharmacology, toxicology and pharmaceutics, and mathematics during 2019–2022. The results suggest that contemporary researchers appear more interested in analyzing medicinal and psychological aspects of cyberbullying and mental health along with developing interests in organizational aspects.

**Table 5:** Research areas.

**Area**	**Overall**	**2019–2022**	**%**	**2010–2018**	**%**
Medicine	294	205	69.73	89	30.27
Psychology	146	102	69.86	44	30.14
Social sciences	117	83	70.94	34	29.06
Environmental science	71	67	94.37	4	5.63
Computer science	39	30	76.92	9	23.08
Neuroscience	20	17	85.00	3	15.00
Arts and humanities	25	12	48.00	13	52.00
Nursing	18	11	61.11	7	38.89
Engineering	12	9	75.00	3	25.00
Multidisciplinary	11	8	72.73	3	27.27
Health Professions	7	5	71.43	2	28.57
Biochemistry, genetics and molecular biology	8	4	50.00	4	50.00
Business, management and accounting	4	4	100.00	0	0.00
Agricultural and biological sciences	6	3	50.00	3	50.00
Energy	3	3	100.00	0	0.00
Pharmacology, toxicology and pharmaceutics	2	2	100.00	0	0.00
Veterinary	2	2	100.00	0	0.00
Economics, econometrics and finance	2	1	50.00	1	50.00
Materials science	2	1	50.00	1	50.00
Mathematics	1	1	100.00	0	0.00

Source: SCOPUS database, % Number of papers published during the period ^*^100/Number of papers published during 2010–2022.

### Topical analysis

**Figure 6:**
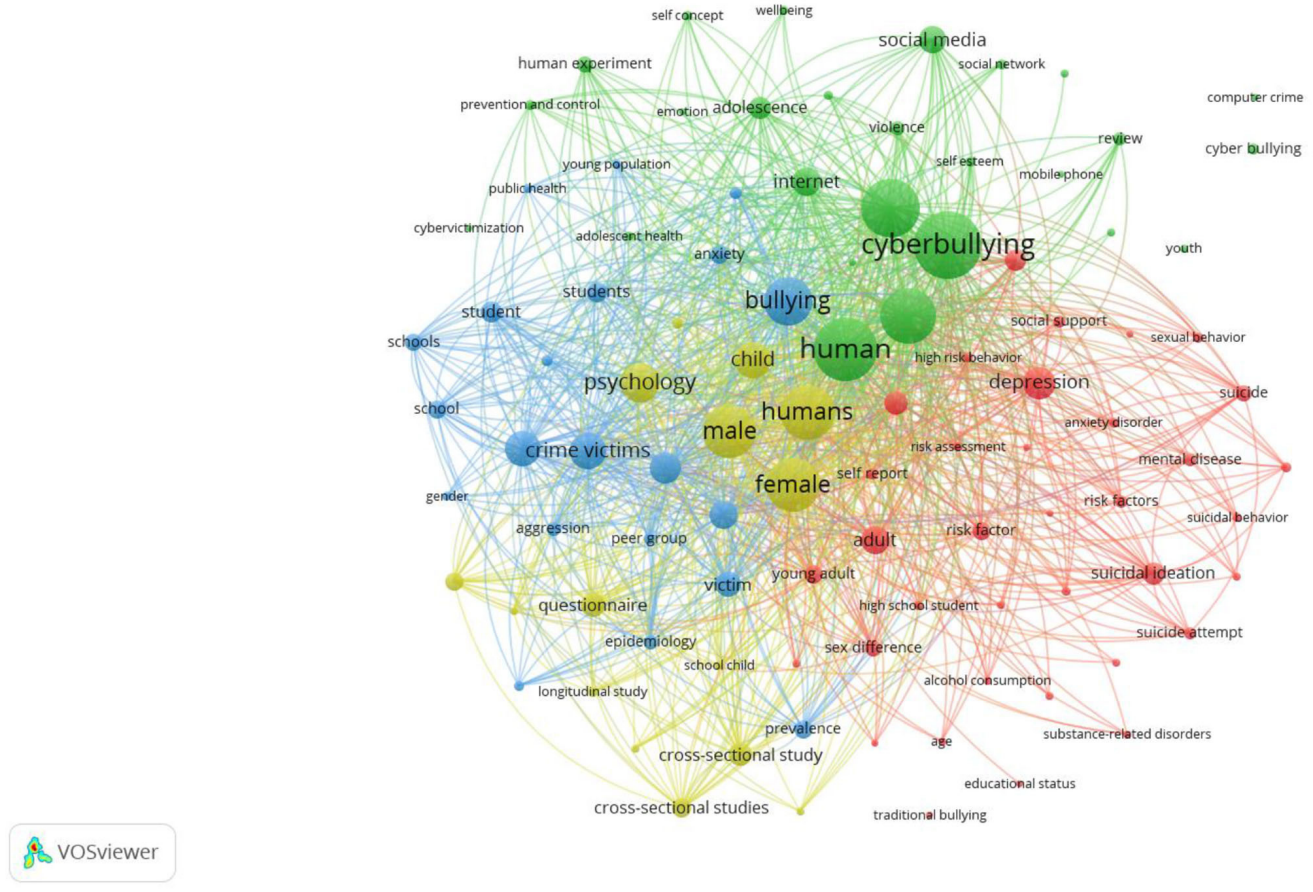
Keyword network visualization. Source: SCOPUS, VOSviewer.

Figure 6 illustrate the topical analysis based on keywords co-occurrence analysis, which suggest that 98 out of 2 420 keyword topics met the threshold criteria of 15 co-occurrences per keyword. The prominent keywords were cyberbullying (353 occurrences) followed by human (324 occurrences), mental health (228 occurrences), adolescent (268 occurrences), and humans (265 occurrences), male (254 occurrences), and female (254 occurrences). The keyword visualization map highlighted five major clusters based on their link to strength co-occurrence.

#### Cluster 1: early studies on cyberbullying and mental health

This cluster (in green color) includes keywords such as cyberbullying, human, mental health, adolescent, internet, prevention and control, adolescent health, etcetera. It appears to comprise early studies that theorized cyberbullying and mental health. For example, Wang et al. (2010) analyzed and recognized cyberbullying as a subtype of bullying. Williams and Godfrey (2011) conceptualized how psychiatric-mental health nurses can recognize cyberbullying. Keung (2011) assessed the relationship between internet addiction and antisocial internet behavior like cyberbullying in adolescents. Goebert et al. (2011) explored the relationship between cyberbullying, substance abuse, and mental health. Additionally, this cluster includes publications studying coping behaviors and the need to develop preventive measures against degraded mental health due to cyberbullying. For instance, school students try to cope with cyberbullying in three ways: reactive, preventive, and in no way to prevent cyberbullying (Parris et al., 2012). Reactive coping strategy may involve ignoring or deleting bullying messages, while preventing coping strategies may involve seeking help or increasing awareness about their security. The study further highlighted that when these strategies fail, students feel defenseless and that there is no way to reduce cyberbullying. Furthermore, Sampasa-Kanyinga and Hamilton (2015) highlighted the need to develop preventive interventions against cyberbullying. Their study suggested that cyberbullying victimization mediated the relationship between the use of social networking sites (SNS), psychological distress and suicidal ideations.

#### Cluster 2: gender in cyberbullying and mental health

The yellow-colored cluster contains keywords such as humans (265 occurrences), female (254 occurrences), male (254 occurrences), psychology (167 occurrences), child (147 occurrences), sex factors (20 occurrences), etcetera. It seems to present studies on gender-based differences in cyberbullying and mental health, often referred to as the gender debate cluster. This cluster contains studies contributing to the ongoing gender-based debate on cyberbullying and mental health. Various researchers have presented differing views on gender-based differences affecting cyberbullying and mental health. For example, Bannink et al. (2014) suggested that male participants showed resilience toward cyberbullying victimization, thereby not developing any mental health problems, whereas it was the opposite in the case of females. Merrill and Hanson (2016) also suggested higher cyberbullying victimization in female participants than in males, and that females sometimes bully other females as a way to release their mental pressure and emotional bursts. On the other hand, Kim et al. (2019) suggested a higher direct effect of cyberbullying victimization on depression in males compared to females. Hood and Duffy (2018) contributed to this debate by revealing that gender differences did not affect the cyberbullying intentions of adolescents. Fletcher et al. (2014) also reported no association of gender with cyberbullying in school learners. Additionally, this cluster consists of longitudinal studies that examine the relationship between cyberbullying and mental health over time. For instance, one of these longitudinal studies predicts cyber victimization in year one followed by developing anxiety in the following year. Similar results were predicted for cybervictimization in year two and anxiety in the following year. Another longitudinal study found a significant moderating effect of perceived social support on the relationship between homophobic cyberbullying, depression, and anxiety (Wright et al., 2022).

#### Cluster 3: clinical and criminal studies on cyberbullying and mental health

This cluster in blue color illustrates studies related to clinical assessments and criminology on cyberbullying and mental health. Some of the keywords are bullying (219 occurrences), crime victims (147 occurrences), major clinical studies (105 occurrences), offender (24 occurrences), etcetera. One of the studies in the field of criminology suggested no significant differences in psychosomatic issues between victims of cyberbullying and traditional bullying (Beckman et al., 2012). Paat and Markham's (2020) review study highlighted the modern-day cyber risks teenagers face, particularly focusing on the hidden dangers associated with cyberbullying, SNSs, cyberdating, and sexting, aiming to raise awareness about these crimes. Another clinical study analyzed the frequency of cyberbullying as a contributing factor to youth suicide in Canada (Cénat et al., 2015). Though cyberbullying was not directly associated with suicide-related deaths, the presence of other mental health issues combined with traditional bullying and cyberbullying contributed to a higher prevalence of identified mental health issues, increasing the risk of suicide attempts and, in some cases, resulting in death (Cénat et al., 2015). Patchin and Hinduja (2017), two prominent authors in the field of cyberbullying, conducted a study to analyze the prevalence of digital self-harm in adolescents. They suggested that males were more likely to engage in digital self-harm activities than females. They also highlighted various factors including sexual orientation, experience with cyberbullying and depressive symptoms which significantly correlated with digital self-harm in adolescents.

#### Cluster 4: associating cyberbullying with mental health

This cluster in red color presents studies that have expanded the literature on cyberbullying and mental health. These studies have used more robust statistical methods to study various variables' moderation and mediation effects on the relationship between cyberbullying and mental health. Some of the keywords present in this cluster are depression (127 occurrences), adult (106 occurrences), and social support (33 occurrences). Feinstein et al. (2014) suggested that rumination significantly mediated the relationship between cyberbullying victimization and depressive symptoms in women. Elgar et al. (2014) examined the importance of family dinners, specifically family communication and contacts, in mitigating the negative impacts of cyberbullying victimization on mental health. They suggested that improved family communication helps alleviate mental stress and reduces suicidal tendencies, depression, and anxiety in cyberbullying victims. Sampasa-Kanyinga and Hamilton (2015) highlighted the need to explore the use of social networking sites in light of cyberbullying victimization to prevent adolescents' mental health issues. They also suggested that cyberbullying victimization mediates the linkages between social networking sites usage and mental health issues such as psychological distress and suicide attempts. Yu and Chao (2016) suggested that internet addiction significantly moderates the relationship between cyberbullying, cyber-pornography, and mental health. They suggested that parents and academics regulate the digital behavior of adolescents and guide them to use the internet better. Lin et al. (2022) indicated that people with higher resilience scores are well protected against depression resulting from cyberbullying victimization. Hence, studies clearly showed the importance of studying individual-level interventions to protect against cyberbullying and resulting mental issues.

### Recent trends

**Figure 7:**
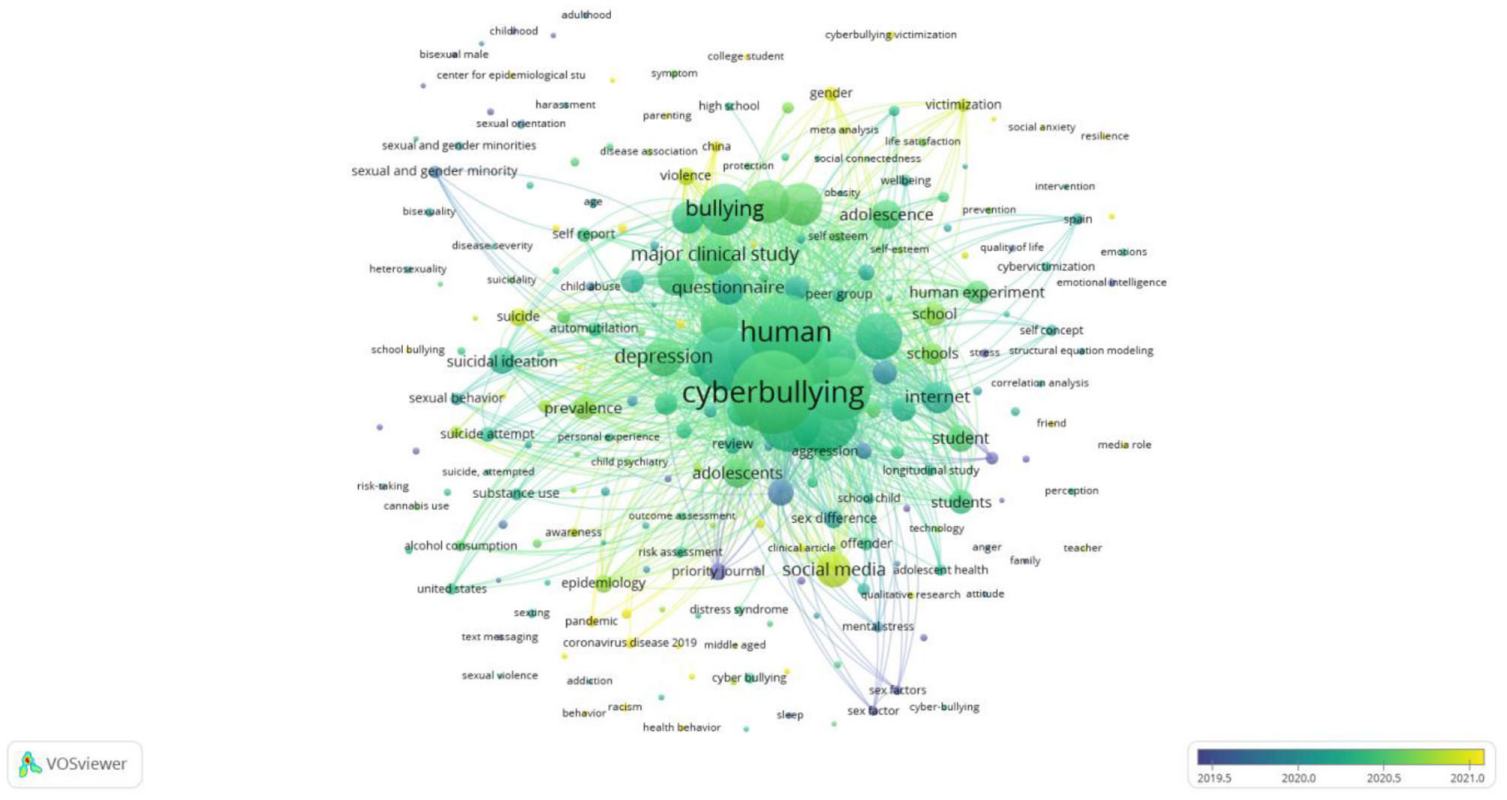
Keyword overlay map. Source: SCOPUS, VOSviewer.

Figure 7 represents the keyword overlay map of emerging topics and evolution/shifts in topics during the last 5 years, i.e., 2018–2022. Again, clusters are formed but based on the time of occurrence of keywords. Keywords presented in yellow and light-green colors indicate the emerging topics.

#### Cluster 1: most emerging cluster

This yellow-colored cluster presents the most emerging topics on cyberbullying and mental health. This cluster has keywords such as social anxiety, racism, pandemics, physiological distress, COVID-19, college students, workplace, etc. According to Hou et al. (2022), there has been an increase in cyberbullying cases due to increased screen time during the COVID-19 pandemic. Studies on college students suggest that the unguided authority to surf the internet has made the collegiate to fall prey to cyberbullies or, in some cases, become one, knowingly or unknowingly (Naik et al., 2021). Bansal et al. (2023a) also reported a positive correlation between depressive symptoms and cyberbullying perpetration in college students. Moreover, Baiden et al. (2022) and Cheah et al. (2020) indicated that racial minorities experience online discrimination, which contributes to increased mental health issues. Cheah et al. (2020) further recommended that healthcare professionals pay special attention to victims of online discrimination during COVID-19.

#### Cluster 2: second most emerging cluster

This parrot-green colored cluster includes studies on the second most emerging topics. It contains keywords such as social media, prevalence, violence, epidemiology, suicide, and gender. For example, Tamarit et al. (2021) explored the relationship between social network sites' addiction, self-esteem and sexo-erotic risk behavior like sexting and grooming in adolescents. Their findings also suggest that online addiction predicts sexo-erotic behavior, and such behavior is moderated by self-esteem. Rakic et al. (2021) suggest that individual and family factors including gender and family affluence status predict cyberbullying exposure in Serbian school students. Marengo et al. (2021) examined the occurrences of cyberbullying and problematic social media use in school students. They found that the risk of being cyber-victimized was higher when problematic social media use was present, and the reports of such cases were higher in female students than in males.

#### Cluster 3: interventions for cyberbullying and mental health

This cluster in teal color contains studies on preventing the degradation of mental health due to cyberbullying. This cluster contains words such as life satisfaction, prevention, mental health, cybervictimization, interventions, etcetera. Several studies have discussed interventions aimed at preventing cyberbullying and promoting sound mental health. For instance, Yosep et al. (2022) and Berardelli et al. (2018) explored and reviewed the literature on interventions that can decrease cyberbullying incidences and mitigate its negative effects on mental health. Yosep et al. (2022) suggested that nursing interventions such as prevention activities, peer-group support, and resilience programs can assist to reduce the occurrence of cyberbullying incidences and help improve mental health. Further, Berardelli et al. (2018) found that lifestyle behavior including cyberbullying, substance abuse, low exercise, and poor diet can severely affect mental health. They indicated that community-wide programs such as social skills training and psychoeducational family treatments can act as interventions against such behaviors and help elevate mental health. Myers and Cowie (2019) reviewed the fragmented literature on cyberbullying victimization in educational institutions and suggested some helpful interventions. They emphasized that educational institutions might adopt social and emotional learning programs to enhance emotional intelligence and empathy among students. The authors also highlighted Smith et al.'s (2016) restorative methods to create a co-operative and positive environment in schools, fostering positive relationships and bolstering the participation of academics and students in implementing such methods.

#### Cluster 4: adolescents and research techniques

This bluish-purple cluster presents studies on adolescent behavior and data gathering techniques. Keywords include adolescent behavior, surveys and questionnaires, psychological wellbeing, priority journal, internet addiction, mental disorders, sexual and gender minority. Various researchers including Barlett et al. (2016), Cavalcanti et al. (2019), Bansal et al. (2022), Tanrikulu and Erdur-Baker (2021) and Reif et al. (2021) have explored the psychometric properties of various scales or inventories measuring cyberbullying in countries like the USA, Brazil, India, Turkey, Spain and Germany, respectively. Studies within this cluster also investigate and suggest the associations between cyber intimate partner victimization and alcohol use (Trujillo et al., 2020), the relationship between cyberbullying experiences, gender, and depression (Alrajeh et al., 2021). Additionally, authors like Reed et al. (2019) suggest that cyber sexual harassment from unknown males is prevalent among teenage girls and negatively affects their mental wellbeing. Some participants from their study also reported the prevalence of cyber sexual harassment from known males.

## Discussion

Studies have shown that cyberbullying has severely affected both the victims' and bullies' physical and mental wellbeing (Kowalski et al., 2014; Bansal et al., 2023a). For instance, incidences of cyberbullying have led to the development of psychological issues such as depression (Litwiller and Brausch, 2013), and suicidal thoughts (Islam et al., 2021). Therefore, numerous researchers have made significant contributions to the literature in order to deepen our understanding of the phenomenon and to explore various aspects, including prevention and interventions. Consequently, a vast body of knowledge has been generated, examining cyberbullying from different perspective such as economics, adolescents, and mental health. This vast literature has provided valuable insights through literature reviews, systematic literature reviews, meta-analyses, and bibliometric analyses. Although these studies have deepened our understanding, they have their own limitations. Hence, this study analyzed the academic performance of research articles through a qualitative bibliometric analysis of cyberbullying and mental health. It explores the development of knowledge in the field of cyberbullying and mental health. Lastly, this study was based on the suggestions and guidelines provided by Donthu et al. (2021) in relation to identifying trends in a field of study through the analysis of published literature.

The very first question answered was based on analyzing the publication and citation trends. Initially, there were < 10 publications per year on the link between cyberbullying and mental health, which increased over a period of time. Similarly, the cumulative citations have also increased to 12,322 by 2022. There could be various reasons for these results. During the initial years of research on cyberbullying, researchers focused on understanding the phenomenon of cyberbullying (Kowalski et al., 2014). Also, during those years, present-day ICT infrastructure was developing, and few people had access to such infrastructure. After the advancements in SNS and ICT, the world saw a boom in ICT (Yang and Hu, 2019). Furthermore, the rise of social media, attributed by the availability of ICT tools and wider accessibility of internet services, made many people spend their time on online activities. This rendered an opportunity for the cyberbullies to deter their acts (Chan et al., 2019). This trend was fuelled further by the COVID-19 pandemic. Restrictions on social and outdoor activities and the shift of study and workplace from offline to online during the pandemic caused people to increase their screen time (Singh et al., 2021). Due to increased screen time, people fell prey to the hands of cyber bullies, especially the younger generation (Bansal et al., 2023a). Zhu et al. (2021) and Han et al. (2021) state that cyberbullying during COVID-19 also contributed to increased mental health issues, including anxiety and depression. These reasons supported the advancements in the research from understanding the phenomenon of cyberbullying to studying its consequences, specifically understanding its impact on the mental health of its victims and perpetrators (Kowalski et al., 2018).

Next, a journal analysis was conducted. The most influential journals and emerging journals were identified. The researchers aimed to determine a shift in the publication area of these identified journals. The Journal of Adolescents Health ranked first with 2 016 citations, followed by the International Journal of Environmental Research and Public Health (703 citations) and the Children and Youth Services Review (535 citations). With the help of overlay analysis, the emerging journals were identified and marked in yellow. The American Journal of Health Promotion, Australian Psychiatry, BMC Psychology, and Frontiers in Digital Health are some of the emerging journals. The overlay analysis was also used to explore the shift in the preferred research areas which journals publish. The earlier journals, like the International Journal of Environmental Research and Public Health, focused on publishing studies related to cyberbullying, the psychological wellbeing of children and youth, and social work practices. The studies published in these journals analyzed cyberbullying not only as a single but also explored its associations with other fields, including medicine and social sciences. This made these fields the best possible fields to conduct research on cyberbullying. Hence, the multidisciplinary nature of these journals confirms that cyberbullying is a multidisciplinary phenomenon with vast future potential for researchers.

In 2018, the focus shifted to the emotional wellness of children and psychological perceptions of using computers and electronic devices. In 2018, influential journals were IEEE Access, Clinical Child Psychology and Psychiatry. The current focus is on neuropsychology, health-cultural-environmental communications, and clinical developments in psychology. Emerging journals are Current Psychology, BMC Psychology and Frontiers in Communication, to name a few. The literature from these journals will help authors identify trends in the research and new research avenues. The journals focused on publishing studies based on the wellbeing of children and youth and social work practices among them. The focus shifted to studies exploring human interaction with electronic devices. Finally, the current focus is on fields such as personality, positive psychology, and neuropsychology. Further, the analysis of the most influential articles revealed that articles titled “Psychological, physical, and academic correlates of cyberbullying and traditional bullying” by Kowalski and Limber (2013); “Cyberbullying, School Bullying, and Psychological Distress: A Regional Census of High School Students” by Schneider et al. (2012); and “Cyber bullying and physical bullying in adolescent suicide: the role of violent behavior and substance use” by Litwiller and Brausch (2013) are among the top ten most influential articles on the topic. The authors studied these articles to gain a better understanding of the topic.

The most influential publications on the topic were explored. It was found that Kowalski and Limber's (2013) article topped the list. It was published in the Journal of Adolescent Health and had received 682 citations until the date of data extraction. These authors studied the nexus between cyberbullying and traditional bullying experiences and their impact on the mental health of children and adolescents. This article was followed by “Cyberbullying, School Bullying, and Psychological Distress: A Regional Census of High School Students.” The article was published in the American Journal of the Public Health and was authored by Schneider et al. (2012). The authors proposed a relationship between psychological distress and the instances of school and cyberbullying. The results of the document analysis also revealed that three out of the top 10 most influential publications were published by the Journal of Adolescent Health. Additionally, a document citation analysis on the data from the past 5 years was conducted. A document titled “Annual Research Review: The persistent and pervasive impact of being bullied in childhood and adolescence: implications for policy and practice” ranked first. The document was published in the Journal of Child Psychology and Psychiatry and was cited 192 times. The authors assessed the impacts of bullying on physical and mental health of children and adolescents.

The publications were then analyzed per country, and emerging countries in the field of study were identified. The results suggest that cyberbullying and mental health have attracted global attention and are not limited to a particular nation. In terms of publication trends, the USA ranked the highest among developed countries, followed by the UK and Spain. Researchers have attributed early research trends in developed countries to technological advancements and early adoption of ICT by citizens (Englander, 2021), thereby increasing time spent on online activities and, consequently, an increase in cyberbullying (Kowalski et al., 2022). Among developing countries, the People's Republic of China took the lead, followed by Indonesia and Vietnam. The analysis also reported that the most influential authors on the topic are from developed nations. P. K. Smith topped the list. Smith et al. (2008) also formulated the widely accepted definition of cyberbullying as defined earlier in this article. However, the overall country analysis revealed confusion as to why the two most populous nations, India, and China, have fewer publications than developed countries on the subject matter. To answer this question, a country overlay analysis was conducted. As expected, these two nations and other developing countries were marked in yellow, indicating their status as emerging countries that will likely play a leading role in future research on cyberbullying and its effects. Future researchers can focus on these emerging countries as most of these are Asian countries and regions which can provide deeper and a different understanding of cyberbullying and mental health due to rich cultural and religious differences from the western nations that may impact the development of attitudes and the behavior of residents of these Asian countries and regions.

Finally, a topical analysis was conducted to identify emerging keywords/topics in this field. The results suggest that cyberbullying and mental health are multidisciplinary. Words such as cyberbullying, human, mental health, psychology, child, adolescent, crime victims, major clinical studies, victims, crime victims, depression, victimization, gender, prevention, risk factors, interventions, COVID-19 and bullying are some of the widely used keywords. Based on the keyword analysis, four differently colored clusters comprising various keywords were retrieved. Cluster 1, in green, illustrated early studies on cyberbullying and mental health. A few of the keywords are cyberbullying, human and mental health. Cluster 2, in yellow, presents studies on gender in cyberbullying and mental health. Some keywords are humans, female, male and sex factors. Cluster 3, in blue, contains clinical and criminal studies on the topic. A few keywords are bullying, crime victims, major clinical studies and offender. Cluster 4, in red, presents studies that found linkages between cyberbullying and mental health. A few of the keywords are depression, adult, and social support. Lastly, the emerging trends were analyzed based on the usage of keywords for the period 2018–2022.

Cluster 1, in yellow, presents the most emerging topics in the cyberbullying and mental health domain. A few of the keywords used are social anxiety, racism, pandemics, COVID-19 and college students. Cluster 2, in parrot green, presents this domain's second most emerging topic. A few of its keywords are social media, violence, and epidemiology. Cluster 3 in teal color presents topics on interventions for cyberbullying and mental health. A few of its keywords are satisfaction, prevention, and intervention. The final Cluster 4, in bluish-purple, contains topics related to adolescents and research techniques. A few of its keywords are adolescent behavior, surveys, and questionnaires. Based on the emerging trends, Cluster 1 in yellow contains emerging keywords that have recently been used, whereas the opposite is true for Cluster 4 in bluish purple.

### Implications of the study

The present study analyses the academic performance of the research articles and sets the future agenda for researchers in the cyberbullying and mental health domain. The study is not limited to a geographical location, culture, institutional setup, or age group. Hence, it provides an overall view of the topic of cyberbullying and mental health. Firstly, the results from first question presented the historic and current performance of the literatures. It further indicated a rising trend in publications and citations, which suggested a great future and increasing importance of the topic. These results will assist researchers by making them aware that the research potential of the topic is vast and increasing, as depicted by publication and citation trend analysis. Secondly, it analyses the most active and emerging countries publishing on the domain. In other words, the results indicated countries where the research has started but is in a nascent stage and countries where the research has not been started and requires focus. Thirdly, it helps researchers recognize a shift in the area of publication of journals. Fourthly, it helps them identify the most influential journals and articles on the topic of cyberbullying. For instance, the results presented a list of top 10 seminal articles and a list of top five emerging articles that can become a strong base of literature for the stakeholders. Lastly, by providing the topical analysis, this study informs the researchers about the emerging themes on the topic. For instance, future researchers can conduct a bibliometric analysis of cyberbullying from the perpetrator's angle. This study encourages researchers to explore the impact of cyberbullying on the mental health of people. Significant progress has been made globally in understanding cyberbullying. Now it is time to understand its impact of cyberbullying on people's mental health and to devise measures to reduce the impact. Hence, the study will help researchers analyze the future agendas on cyberbullying and mental health and to determine research gaps that require in-depth studies. The study will also help policymakers and academics to develop programs and policies for creating awareness regarding cyberbullying and its impact on mental health at both academic and other institutional levels. Programs on the topic will also help people understand the phenomenon of cyberbullying and reduce the feelings that hamper them from asking for help if trapped in cyberbullying or experiencing its adverse impact on their mental health. Furthermore, the laws in India do not adequately describe cyberbullying and its consequences. Hence, the definitions of cyberbullying offered in this study, especially by Smith et al. (2008), will help the Indian policymakers to define cyberbullying, identity it as a crime, and frame rules/corrections/punishments based on severity depending on the intensity of consequences its victim suffers. For instance, a victim committing suicide after experiencing cyberbullying may be deemed as the most severe consequence of the cyberbullying and the law makers may define rules accordingly.

### Limitation, scope for future studies, and conclusion

The study provides exciting results regarding the academic performance of the literature on cyberbullying and mental health. It still has certain limitations. Peer-reviewed journal articles were considered whilst excluding other sources such as book titles and articles presented at research conferences. The search was limited to articles in the English language only. Although English is considered lingua franca in academia, many authors prefer publishing their works in their mother tongues. Finally, data were extracted from a single database, namely SCOPUS. This may raise questions about research bias. It is suggested that future researchers consider these points and enhance the bibliometric base on the topic.

## Conclusion

To conclude, this study analyzed academic performance of research articles on the topic of cyberbullying and its impact on people's mental health and explored the future research agenda on cyberbullying and mental health. The study answered questions such as the following: (a) What is the current trend in the publication and citations of research articles? (b.1) What is the current academic performance of countries based on their research article publications? (b.2) Which are the emerging countries and the origin countries of the most influential authors? (c) What are the most influential journals, and what is the trend of publication based on journals' preferred publication domains associated with the research topic? (d) Which are the most influential articles on the topic under investigation? (e) What are the emerging topics related to cyberbullying and mental health?

## Data availability statement

The original contributions presented in the study are included in the article/supplementary material, further inquiries can be directed to the corresponding author/s.

## Author contributions

SB: Conceptualization, Data curation, Formal analysis, Funding acquisition, Investigation, Methodology, Project administration, Software, Supervision, Writing – original draft. NG: Conceptualization, Data curation, Formal analysis, Investigation, Methodology, Project administration, Writing – original draft, Writing – review & editing. JS: Resources, Validation, Visualization, Writing – original draft. FV: Formal analysis, Funding acquisition, Writing – review & editing.
